# Virtual Reality–Based Psychotherapy in Social Anxiety Disorder: fMRI Study Using a Self-Referential Task

**DOI:** 10.2196/25731

**Published:** 2021-04-14

**Authors:** Ji-Won Hur, Hyemin Shin, Dooyoung Jung, Heon-Jeong Lee, Sungkil Lee, Gerard J Kim, Chung-Yean Cho, Seungmoon Choi, Seung-Moo Lee, Chul-Hyun Cho

**Affiliations:** 1 School of Psychology Korea University Seoul Republic of Korea; 2 Department of Biomedical Engineering Ulsan National Institute of Science and Technology Ulsan Republic of Korea; 3 Department of Psychiatry Korea University College of Medicine Seoul Republic of Korea; 4 Department of Software Sungkyunkwan University Suwon Republic of Korea; 5 Digital Experience Laboratory Department of Computer Science and Engineering Korea University Seoul Republic of Korea; 6 Department of Film & Multimedia Korea National University of Arts Seoul Republic of Korea; 7 Department of Computer Science and Engineering Pohang University of Science and Technology Pohang Republic of Korea; 8 Department of Psychiatry College of Medicine Chungnam National University Daejeon Republic of Korea; 9 Department of Psychiatry Chungnam National University Sejong Hospital Sejong Republic of Korea

**Keywords:** virtual reality, VR, social anxiety, social phobia, exposure therapy, fMRI, unctional magnetic resonance imaging

## Abstract

**Background:**

Although it has been well demonstrated that the efficacy of virtual reality therapy for social anxiety disorder is comparable to that of traditional cognitive behavioral therapy, little is known about the effect of virtual reality on pathological self-referential processes in individuals with social anxiety disorder.

**Objective:**

We aimed to determine changes in self-referential processing and their neural mechanisms following virtual reality treatment.

**Methods:**

We recruited participants with and without a primary diagnosis of social anxiety disorder to undergo clinical assessments (Social Phobia Scale and Post-Event Rumination Scale) and functional magnetic resonance imaging (fMRI) scans. Participants with social anxiety disorder received virtual reality–based exposure treatment for 6 sessions starting immediately after baseline testing. After the sixth session, participants with social anxiety disorder completed follow-up scans during which they were asked to judge whether a series of words (positive, negative, neutral) was relevant to them.

**Results:**

Of 25 individuals with social anxiety disorder who participated in the study, 21 completed the sessions and follow-up; 22 control individuals also participated. There were no significant differences in age (*P*=.36), sex (*P*=.71), or handedness (*P*=.51) between the groups. Whole-brain analysis revealed that participants in the social anxiety disorder group had increased neural responses during positive self-referential processing in the medial temporal and frontal cortexes compared with those in the control group. Participants in the social anxiety disorder group also showed increased left insular activation and decreased right middle frontal gyrus activation during negative self-referential processing. After undergoing virtual reality–based therapy, overall symptoms of the participants with social anxiety disorder were reduced, and these participants exhibited greater activity in a brain regions responsible for self-referential and autobiographical memory processes while viewing positive words during postintervention fMRI scans. Interestingly, the greater the blood oxygen level dependent changes related to positive self-referential processing, the lower the tendency to ruminate on the negative events and the lower the social anxiety following the virtual reality session. Compared with that at baseline, higher activation was also found within broad somatosensory areas in individuals with social anxiety disorder during negative self-referential processing following virtual reality therapy.

**Conclusions:**

These fMRI findings might reflect the enhanced physiological and cognitive processing in individuals with social anxiety disorder in response to self-referential information. They also provide neural evidence of the effect of virtual reality exposure therapy on social anxiety and self-derogation.

## Introduction

Social anxiety disorder (also known as social phobia) is characterized by a persistent fear of social situations in which the person would be exposed to possible scrutiny by others [1], with a lifetime prevalence of 2% to 7% in adults and a prevalence up to 25% in university students [2]. Individuals who have social anxiety disorder are typically hypervigilant for real or imagined feedback and more likely to rely on safety behaviors, aiming to avoid any stimuli or events that could trigger their social anxiety [3,4].

An elaborate cognitive–behavioral model proposed that pathological self-referential processing is an important factor in the developing and continuing to experience social anxiety disorder [5]. Increased activity in cortical midline structures (eg, medial prefrontal cortex, posterior cingulate cortex) and limbic areas (eg, amygdala, anterior cingulate cortex, insula) has been linked to biased self-referential processing [5,6] in social anxiety disorder. Altered activation in these brain areas is also correlated with abnormal self-focused attention [7], which includes socially anxious individuals’ fear of being evaluated [8].

Interestingly, individuals with social anxiety disorder seem to be vulnerable to any type of evaluation toward them. That is, they are sensitive to potential negative evaluations from others (fear of negative evaluation), but also, positive evaluations from others [9] given that a positive social reputation inevitably elevates the individual’s social status, which brings more scrutiny. Empirical evidence indicates that receiving positive social feedback is a horrific experience for patients with social anxiety disorder [10,11]. In addition, it has been shown that the higher the fear of positive evaluation, the higher the social anxiety and discomfort and the lower the assertiveness and perceived accuracy of the feedback [10,12]. Findings on the fear of evaluation in social anxiety disorder have been paralleled by a growing interest in their neurophysiological mechanisms. Previous studies [12] have suggested that aberrant neural activity may exists when individuals with social anxiety disorder receive feedback. Research using positive referential processing has also shown higher activation in bilateral medial prefrontal and inferior frontal cortices, fusiform gyrus, thalamus, left posterior superior temporal gyrus [13], and left posterior insula [14] in social anxiety disorder.

Unfortunately, social anxiety disorder affects self-concept and individual performance directly and long-term [15,16]. Therefore, timely diagnosis and intervention are necessary for those affected by social anxiety disorder. A large body of literature has shown that psychoeducation, imaginal and in vivo exposure, and assertive training can reduce social anxiety symptoms and hyperactivation in brain areas in social anxiety disorder [17-19]; however, a majority of individuals do not place social phobia as a priority for treatment, as they become distracted by other coexisting diseases or regard their symptoms as an inherited temperament such as shyness [20].

In recent years, virtual reality (VR) therapeutics, developed to overcome restricted accessibility to conventional psychotherapies, have been widely used as a valid and effective platform for patients with social anxiety disorder to learn evidence-based coping skills [21,22]. Meta-analyses [23,24] have shown a large effect size of VR exposure therapy for social anxiety disorder and performance anxiety; however, the extent of the effectiveness of social anxiety disorder–specific VR therapies on self-referential processing remains an open research question. Moreover, neurobiological evidence regarding VR-based interventions for social anxiety disorder is still lacking.

In this study, we aimed to assess positive and negative self-referential processing in individuals diagnosed with social anxiety disorder and to explore the effects of VR therapy on neural substrates related to self-referential processing. First, we hypothesized that alterations in self-referencing occur in social anxiety disorder. Second, we speculated that neuronal changes in certain brain regions occur during self-referential processing in individuals with social anxiety disorder who have undergone VR therapy. To assess this, we used VR therapy [25], an alternative to exposure therapy for individuals with social anxiety disorder that provides psychoeducational intervention tailored to social anxiety symptoms. In short, we expected to provide neural evidence of the efficacy and applicability of a VR-based therapeutic approach to alter self-referential processing in individuals with social anxiety disorder.

## Methods

### Recruitment

We recruited individuals with and without social anxiety disorder via advertisements posted online (eg, forums, social media, and a website). Participants with social anxiety disorder were eligible if they met the Diagnostic and Statistical Manual of Mental Disorders, Fourth Edition criteria for social anxiety disorder, which was assessed with the Mini-International Neuropsychiatric Interview [26], and if they had a score ≥82 on the Korean version of the Social Avoidance and Distress Scale.

The exclusion criteria for all participants were (1) having a lifetime or current mental illness or neurological disorder that might elicit severe side effects from a VR experience (eg, schizophrenia spectrum disorder, bipolar disorder, posttraumatic stress disorder, panic disorder, substance use disorders, autism spectrum disorder, epilepsy, traumatic brain injury, suicide attempts); (2) having an intellectual disability (IQ <70; estimated with the short version of the Korean Wechsler Adult Intelligence Test Fourth Edition [27]); and (3) receiving psychotropic medication or psychotherapy at the time of research enrollment.

After they had been given a detailed explanation of the study, all participants provided written consent and completed MRI scanning safety eligibility screen.

### VR-Based Psychotherapy

#### Composition and Contents of the Participatory VR Therapy Program

Individuals diagnosed with social anxiety disorder were asked to participate in the VR therapy program developed and verified by the authors [25]. Two psychiatrists and two licensed psychologists performed collaborative work to develop a social-anxiety scenario (during a team meeting in class), and the Art & Technology Lab at the Korean National University of Arts designed VR environments using the Unity game engine (Unity Technologies). Motion capture was conducted using Sensor Suite (Rokoko Electronics) for VR character animation, and voice acting was performed by 4 actors from Korean National University of Arts drama school. In our previous work [25], we proved that our participatory and interactive VR intervention had significant beneficial effects on depression, anxiety, shame, rumination, and social phobia.

The participatory VR intervention for social anxiety symptoms consisted of 3 stages (introduction, core, and finishing) and was divided into 3 levels of difficulty (easy, medium, and difficult). All participants used a VIVE (HTC Corporation) VR headset, and the participants’ heart rates, skin galvanic response, and eye movements were measured during the VR experience.

In the introduction stage, the participants were invited to choose their avatar to learn how to use VR, calm their minds, and help them adapt to VR during the meditation-based warm-up. A voice guide was provided to help the participants relax and breathe while observing trees gently shaking to calm their minds. Through this, the participants not only adapted to the VR system but also moved to the next stage in a stable state of mind. The introduction stage was configured to take approximately 5 minutes.

The core stage was designed to provide a solution to individuals’ exposure and participation in social anxiety situations by providing them with a VR environment in which a college student group was meeting for the first time to discuss an assigned task and introduce themselves. In the virtual setting, 7 to 8 nonplayer characters introduced themselves then the participants with social anxiety disorder were asked to introduce themselves. At the easy level, each participant took turns introducing themselves in a normal and calm manner, starting with the nonplayer characters. In the difficult situation of encountering an unfavorable reaction from nonplayer characters, the nonplayer characters became increasingly distracted while listening to the participant’s self-introduction, making more small talk among themselves, concentrating harder on other tasks, or staring intently at each other. The medium level consisted of distractions between the easy and difficult levels. The core stage was configured to take approximately 7 to 8 minutes.

In the finishing stage, the participant, as in the introduction stage, once again experienced meditation-based VR that calmed the mind while observing a gently shaking tree and controlling their breathing. The VR program ended by providing general cognitive and behavioral psychoeducation for social anxiety disorder in both voice and text form via the VR system. The final stage was configured to take approximately 3 minutes.

#### Number of Participatory VR Solution Sessions and Rules

All participants were asked to complete a total of 6 VR sessions, each consisting of the 3 stages. In one visit, each participant could complete up to 2 VR sessions as long as the participant took at least a 2-hour break between sessions. All participants started at the easy VR level. From the second session onward, the participants were asked to select the level of difficulty they desired. The difficulty level was increased, maintained, or decreased at the participant's request to provide an individual-tailored intervention. The researchers stayed with the participants throughout the VR sessions to address emergencies such as extreme anxiety or panic attacks.

### Experimental Procedure

#### Overview

All participants underwent fMRI while performing a self-referential processing task and completing self-report questionnaires, including the Korean version of the Social Phobia Scale (SPS) (H. Kim, unpublished) and Post-Event Rumination Scale (PERS) [28]. The participants with social anxiety disorder underwent fMRI and assessments before and after treatment, while the control participants underwent fMRI only once. The recommended sample size for a task fMRI is 20; our sample size (n=25 in the social anxiety disorder group at baseline, n=21 in the social anxiety disorder group at follow-up) seemed to have adequate statistical power [29]. The study was approved by the institutional review board of Korea University Anam Hospital in accordance with the Declaration of Helsinki. This study was registered (Clinical Research Information Service KCT0003854).

#### fMRI Experimental Task

A revised version of the Personal Relevance Rating Task (PRRT) was used for the fMRI task [30]. The task consisted of 2 runs with a duration of 9 minutes 22 seconds per run, and each run included 40 trials (yielding a total of 80 trials). Each stimulus word was projected onto an angled mirror mounted on the head coil for 2 seconds using E-prime software (Psychology Software Tools). Between experimental stimuli, a mask (row of X's; 10.8 seconds) and a fixation cue (1 second; row of X's with prongs around the center X) were presented. The order of presentation of all stimuli was counterbalanced.

To build the set of stimuli, we selected 10 positive words, 10 negative words, and 20 neutral words for the experiment, balanced for arousal level, emotional valence, and word length. In addition to the normed emotional adjectives, 10 positive words and 10 negative words generated by the participants were added to the word list for the fMRI PRRT task. The instructions were as follows: “Generate ten positive words that best represent your strengths” and “Generate ten negative words that best represent your weaknesses.” All of these words in both normed stimuli (10 positive, 10 negative, 20 neutral words) and participant-generated stimuli (10 positive, 10 negative words) were selected from the Korean emotion words list [31].

During the fMRI, participants were instructed to make a judgment on each word as soon as possible by pressing the buttons for “not relevant to me,” “somewhat relevant to me,” or “relevant to me” after the word projected. Prior to the scan, participants completed a practice session to ensure that they understood the task. All responses and reaction times were recorded.

#### Image Acquisition and Analysis

Neuroimaging was performed using an MRI machine (3T Siemens Tim trio) equipped with a 12-channel head coil at the Brain Imaging Center, Korea University. Axial T2*-weighted images (echo time 26 ms, repetition time 2000 ms, flip angle 80°, field of view 210 mm, voxel size 2.5×2.5×3.4, slice thickness 3.4 mm, matrix size 84×84). A single functional run consisted of 278 volumes with 37 sequential axial slices each. In addition, structural T1-weighted images (208 slices; echo time 1.89 ms, repetition time 1670 ms, flip angle 9°, field of view 250 mm, matrix size 256×256) were also obtained to aid with spatial normalization.

Anatomic T1 and functional T2* images were analyzed with Statistical Parametric Mapping software (version 12; Welcome Department of Cognitive Neurology). The echo-planar images were corrected for slice acquisition time and realigned to correct for rigid body transformation, then the individual's anatomic image was coregistered to the mean functional image. The echo-planar images were subsequently normalized using nonlinear transformation parameters obtained by registering individual T1-weighted images to the Montreal Neurological Institute template [32] and smoothed with an isotropic 6-mm^3^ full-width-at-half-maximum Gaussian kernel.

### Statistical Analysis

We compared the baseline for demographic measures of each group using independent *t* tests (sex and handedness) or chi-square tests (age and education). Distributions for all clinical variables were analyzed for normality, skewness, and kurtosis prior to comparative analysis. We compared the baseline measurements of each group using parametric and nonparametric analysis as appropriate. Normally distributed variables (negative PERS score) were analyzed employing an analysis of variance (ANOVA), while variables found to have skewed distributions (SPS, positive PERS scores) were analyzed using Mann–Whitney *U* tests. We also conducted a multivariate analysis of variance (MANOVA) to compare behavioral data between groups. For within-group analysis, ANOVA was used for normally distributed SPS scores to identify differences between baseline and follow-up. Both positive and negative PERS scores before and after the intervention were compared using the Wilcoxon signed-rank test. A repeated measures ANOVA with time (baseline vs follow-up) and valence (positive, negative, neutral) as within-subject factors was used to evaluate changes in behavioral outcomes for participants with social anxiety disorder. To identify the brain regions responsible for positive and negative self-referential processing, first-level contrast images were created using the difference between the blood oxygen level-dependent (BOLD) signals recorded during each condition (positive-word images>neutral-word images; negative-word images>neutral-word images). At the second level, we conducted whole-brain analysis to assess which specific brain regions were sensitive to the conditions between the social anxiety disorder and control groups. Two-sample *t* tests were used to reveal group-related differences in brain activity between the social anxiety disorder and control groups. A paired *t* test was also used to estimate the main effect of the intervention for each condition between baseline and follow-up in the social anxiety disorder group. In the whole-brain analysis, significant activations were reported with an uncorrected threshold of *P*<.001 and a cluster extent threshold of *k*≥20 voxels (equivalent to *t*=3.55), which is recommended to minimize the risk of type I (false-positive) errors [33,34]. This threshold is stricter than the uncorrected *P*<.005, *k*≥20 voxel threshold, which is equivalent to a false discovery rate of .05 [35].

In social anxiety disorder group, the correlations between the percentage signal intensity changes following VR therapy sessions and clinical symptom scores were evaluated using the Spearman correlation coefficient. Statistical analysis of the demographic and clinical data was performed using SPSS Statistics (version 23.0; IBM Corp).

## Results

### Recruitment, Enrollment, and Completion

We initially recruited and enrolled 40 individuals with social anxiety disorder and 33 individuals for the control group who had no other neurological or psychiatric diagnoses; however, from the initial 73 individuals, 23 participants (social anxiety disorder: n=12; control: n=11) were excluded because of missing data (n=14), excessive head motion (n=6), or poor fMRI task performance due to loss of concentration, fatigue, or dizziness (n =3). Data from 4 additional participants were discarded due to head motion (n=1) and declined to be scanned (n=3) at the time of follow-up, and 3 participants with social anxiety disorder dropped out during the study prior completing all 6 sessions. Thus, the data from 25 individuals (15 women) with a primary diagnosis of social anxiety disorder and 22 controls (12 women) were used in the baseline analysis (Table 1), and there were no group differences in age, sex, or handedness. In addition, among the 25 individuals with social anxiety disorder, 21 participants completed the VR session and postintervention assessments.

**Table 1:** Sample characteristics and behavioral data.

Characteristic					Social anxiety disorder (n=25)			Controls (n=22)			Test statistic			*P* value
**Sex, n**											0.14 (1)^a^			.71
	Male			15			12							
	Female			10			10							
Age (years), mean (SD)					23.04 (3.35)			23.95 (3.50)			–0.92 (45)^b^			.36
**Handedness, n**											1.36 (2)^a^			.51
	Left			0			1							
	Right			23			20							
	Both			2			1							
Education (years), mean (SD)					14.44 (1.45)			15.09 (1.88)			–1.34 (45)^b^			.19
Social Phobia Scale, mean (SD)					32.52 (13.17)			6.05 (5.26)			–5.67^c^			<.001
Negative PERS^d^, mean (SD)					32.24 (11.18)			8.18 (6.23)			79.92 (1,45)^e^			<.001
Positive PERS^d^, mean (SD)					13.84 (5.41)			26.64 (9.44)			4.28^c^			<.001
**Personal Relevance Rating Task, mean (SD)**														
	**Personal relevance rating**													
		Positive	1.97 (.57)			2.52 (.41)			14.23 (.24)^f^			<.001		
		Neutral	2.01 (.30)			1.80 (.35)			5.11 (.10)^f^			.03		
		Negative	2.24 (.42)			1.61 (.48)			22.53 (.33)^f^			<.001		
	**Reaction time (milliseconds)**													
		Positive	1567.5 (842.7)			1174.8 (761.4)			2.78 (.06)^f^			.10		
		Neutral	1601.7 (784.9)			1270.2 (547.3)			2.75 (.06)^f^			.10		
		Negative	1539.8 (720.0)			1187.0 (549.0)			3.49 (.07)^f^			.07		

^a^Chi square (*df*).

^b^*t* test statistic (*df*).

^c^Mann–Whitney *U* test *Z* statistic.

^d^PERS: Post-Event Rumination Scale. Task response ratings were scored on a 3-point Likert scale (1=not relevant; 2=somewhat relevant; 3=relevant).

^e^*F*(*df1*,*df2*).

^f^*F*(η^2^_p_).

### Full Sample Description at Baseline

#### Demographic and Clinical Characteristics

Demographics did not differ significantly between the 2 groups (sex, *P*=.71; age, *P*=.36; handedness, *P*=.51; education, *P*=.19), but compared with the controls, individuals with social anxiety disorder had higher SPS (Mann–Whitney *U* test: *Z*=−5.67, *P*<.001, *r*=−.83) and negative PERS (*F*_1,47_=79.92, *P*<.001, η^2^_p_=.64) scores as well as lower positive PERS scores (Mann–Whitney *U* test: *Z*=4.28, *P*<.001, *r*=−.63) (Table 1).

#### Behavioral Outcomes

Statistically significant multivariate effects were found between the social anxiety disorder and control groups for the response ratings and reaction times for words of every valence (positive, neutral, and negative) (Wilks lambda=.58, *F*_3,43_=10.41, *P*<.001, η^2^_p_=.42). Participants who were highly socially anxious rated positive words as less (*F*_1,45_=14.23, *P*<.001, η^2^_p_=.24) and negative words as more personally relevant (*F*_1,45_=22.53, *P*<.001, η^2^_p_=.33) than controls did. There were no significant group differences in reaction time for any valence (Wilks lambda=.93, *F*_3,43_=1.11, *P*=.36, η^2^_p_=.07) (Table 1).

#### Neural Correlates of Positive and Negative Self-Referential Processing at Baseline

**Figure 1:**
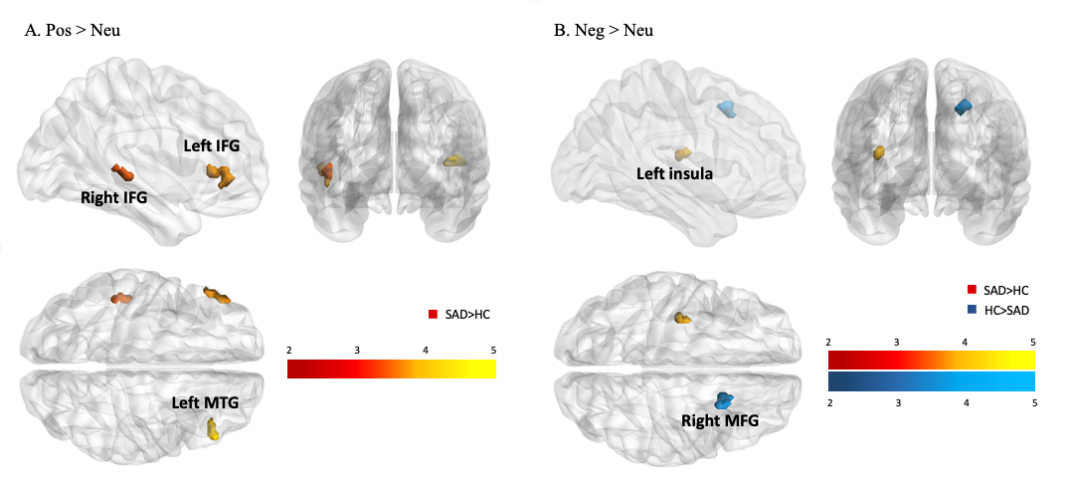
(A) Statistical parametric map showing activation differences between the social anxiety disorder and control groups during positive self-referential processing. (B) Statistical parametric map showing activations associated with negative self-referential processing. HC: healthy control; IFG: inferior frontal gyrus; MFG: middle frontal gyrus; MTG: middle temporal gyrus; Neg: negative words; Neu: neutral words; Pos: positive words; SAD: social anxiety disorder. The color bar depicts Z values.

In the whole-brain analysis, compared with the controls, the social anxiety disorder group showed increased activation of the bilateral inferior frontal gyrus and left middle temporal gyrus during positive self-referential processing (positive-word images>neutral-word images). In individuals with social anxiety disorder, there was also increased left insula activation and decreased right middle frontal gyrus activation in response to negative self-referential processing (negative-word images>neutral-word images) (Figure 1 and Table 2).

**Table 2:** Brain regions showing group differences in response to self-referential processing.

Valence and region				Coordinate							Statistic^a^					Social anxiety disorder with respect to control
				*x*		*y*		*z*		*k*			*Z*_max_			
**Positive>neutral words**																
	**Inferior frontal gyrus**															
		Right pars triangularis	48		34		8		53			4.09		Greater activation		
		Left pars triangularis	–48		38		4		49			3.80		Greater activation		
	Left middle temporal gyrus			–48		–28		0		24			3.52		Greater activation	
**Negative>neutral words**																
	Left insula			–36		–16		14		23			3.66		Greater activation	
	Right middle frontal gyrus			22		14		46		41			4.05		Less activation	

^a^*t*≥ 3.28, *df*=[1.0, 45.0].

### VR-Based Treatment Responses in Social Anxiety Disorder

#### Behavioral Outcomes

Repeated measures ANOVA for the response ratings and reaction times in the social anxiety disorder group (n=21) showed marginally significant changes in the ratings for the positive words (*F*_1,19_=3.85, *P*=.06, η^2^_p_=.16) and negative words (*F*_1,19_=3.77, *P*=.07, η^2^_p_=.16). No statistically significant changes were found for reaction times before and after VR treatment (Table 3).

**Table 3:** Changes in clinical symptoms and behavior performance of participants with social anxiety disorder following VR therapy.

			Baseline (n=21)	Follow-up (n=21)	Test statistic	*P* value
Social Phobia Scale, mean (SD)			30.86 (13.34)	23.52 (12.34)	9.83 (1,20)^a^	.005
Negative PERS^b^, mean (SD)			31.95 (11.66)	21.19 (11.62)	–3.32^c^	<.001
Positive PERS^b^, mean (SD)			13.86 (5.12)	14.62 (7.61)	.32^c^	.75
**Personal Relevance Rating Task, mean (SD)**						
	**Personal relevance rating**					
		Positive	1.93 (.60)	2.17 (.61)	3.85 (.16)^d^	.06
		Neutral	1.98 (.28)	2.05 (.30)	1.18 (.06)^d^	.29
		Negative	2.18 (.41)	2.01 (.42)	3.77 (.16)^d^	.07
	**Reaction time (milliseconds)**					
		Positive	1594.0 (894.8)	1333.7 (458.3)	2.10 (.10)^d^	.16
		Neutral	1677.0 (823.0)	1469.1 (661.0)	1.15 (.05)^d^	.30
		Negative	1599.7 (743.0)	1369.7 (577.9)	1.99 (.09)^d^	.17

^a^*F*(*df1*,*df2*).

^b^PERS: Post-Event Rumination Scale. Task response ratings were scored on a 3-point (1 to 3) Likert scale.

^c^Wilcoxon signed-rank test *Z* statistic.

^d^*F*(η^2^_p_).

#### Changes in Clinical Symptom Severity in Social Anxiety Disorder

The SPS and negative PERS scores of the social anxiety disorder group decreased remarkably (*F*_1,20_=9.83, *P*=.005, η^2^_p_=.33; Wilcoxon signed-rank test: *Z*=–3.32, *P*<.001, *r*=.51, respectively) following VR therapy. There were no significant changes in positive PERS scores between baseline and follow-up (*P*=.75) (Table 3).

#### Changes in Neural Response to Self-Referential Processing in Social Anxiety Disorder

**Figure 2:**
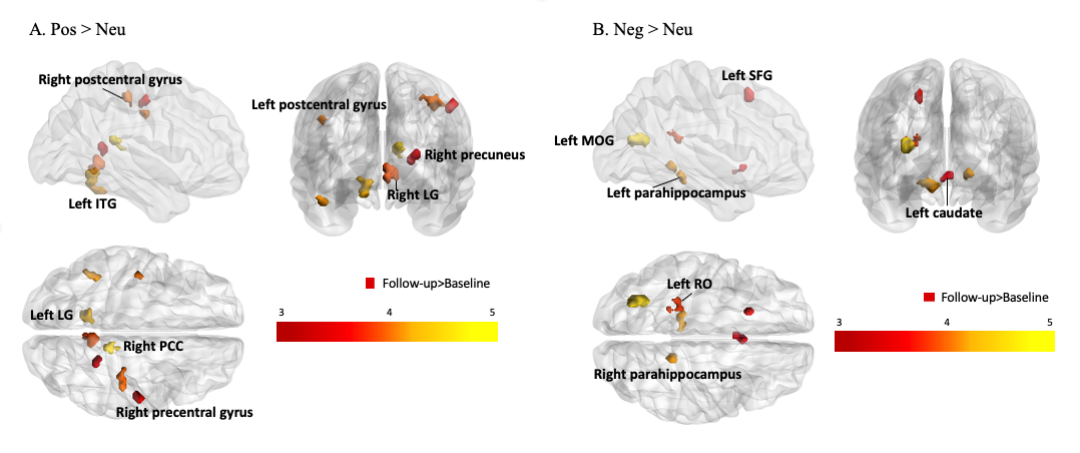
Changes in neural responses during (A) positive self-referential processing and (B) negative self-referential processing among individuals with social anxiety disorder. ITG: inferior temporal gyrus; LG: lingual gyrus; MOG: middle occipital gyrus; Neg: negative words; Neu: neutral words; Pos: positive words; PCC: posterior cingulate cortex; RO: Rolandic operculum; SFG: superior frontal gyrus. The color bar depicts Z values.

The social anxiety disorder group had significantly increased activation of positive self-referential stimuli (positive>neutral) in the right posterior cingulate cortex/precuneus, lingual gyrus, left inferior temporal gyrus, precentral gyrus, and postcentral gyrus after treatment. Moreover, increased activation was found during negative self-referential processing (negative>neutral) in the left middle occipital gyrus, parahippocampus, left Rolandic operculum, left superior frontal gyrus, and left caudate nucleus (Figure 2, Table 4) at follow-up compared with that at baseline.

**Table 4:** Brain regions exhibiting significant changes in self-referential processing in social anxiety disorder following VR therapy.

Valence and region				Coordinate							Statistic^a^					Follow-up with respect to baseline
			*X*		*Y*		*Z*		*k*			*Z*_max_				
**Positive>neutral words**																
	Right posterior cingulate cortex/precuneus			12		–38		18		48			4.38		Greater activation	
	Right posterior cingulate cortex/precuneus			26		–48		12		22			3.45		Greater activation	
	**Lingual gyrus**															
		Left lingual gyrus	–12		–54		–10		57			4.27		Greater activation		
		Right lingual gyrus	6		–50		–2		64			3.96		Greater activation		
	Left inferior temporal gyrus			–46		–50		–24		21			4.13		Greater activation	
	**Postcentral gyrus**															
		Left postcentral gyrus	–44		–12		38		20			4.03		Greater activation		
		Right postcentral gyrus	34		–28		50		69			4.02		Greater activation		
	Right precentral gyrus			52		–14		48		25			3.81		Greater activation	
**Negative>neutral words**																
	Left middle occipital gyrus			–30		–64		16		77			4.40		Greater activation	
	**Parahippocampus**															
		Left parahippocampus	–18		–36		–12		61			4.17		Greater activation		
		Right parahippocampus	16		–42		–4		21			4.10		Greater activation		
	Left Rolandic operculum			–24		–36		18		45			3.95		Greater activation	
	Left superior frontal gyrus			–20		18		52		23			3.88		Greater activation	
	Left caudate nucleus			0		12		–8		37			3.84		Greater activation	

^a^*t*≥3.55, *df*=[1.0, 20.0].

#### Correlation Analyses Between Neural Changes During Self-Referential Processing and Clinical Measures in Social Anxiety Disorder Following VR Therapy

Changes in lingual gyrus activation associated with positive self-referential processing were related to alleviated social anxiety (SPS, Spearman ρ=–.52, *P*=.02) and decreased rumination on the negative events (negative PERS, Spearman ρ=–.61, *P*=.005) in the postintervention sessions. In addition, mean percentage BOLD signal changes in regions revealing significant changes following the VR intervention were not correlated with either positive PERS score at follow-up or the other symptom change rates in social anxiety disorder.

## Discussion

### General

Over the past decade, great endeavors have been made to provide VR-based therapeutic interventions for social anxiety disorder [36]. Individuals with social anxiety disorder continuously allocate their attentional resources to their self-evaluation and self-referential processing. Such cognitive processes can interfere with accurate perception and interpretation of the self and the social environment [37]. These individuals’ excessive self-focused attention will eventually become impediments to social and professional achievements [38]. We aimed to demonstrate the neural correlates of self-referential processing in social anxiety disorder and the changes in brain activations following VR-based therapy, one of the promising interventions for social anxiety. The results demonstrated that individuals diagnosed with social anxiety disorder showed increased neural activity in response to both positive and negative self-referential stimuli. We also found that VR therapy alleviated anxiety symptoms and enhanced neural activity across a wide range of brain areas, including the frontal, temporal, and occipital regions, of individuals with social anxiety disorder. In particular, the SPS scores decreased significantly below the range of severe social anxiety (from mean 30.86, SD 13.34 at baseline to mean 23.52, SD 12.34 at follow-up; the clinical cutoff score for severe social anxiety is 24 [39]). In the correlation analyses, those who showed greater functional changes in the lingual gyrus during positive self-referential processing were observed to have less social anxiety and less engagement in negative rumination in the postintervention sessions. Our results might provide several insights regarding self-referential processing in social anxiety disorder in light of the legacy accumulated by brain imaging studies.

### Increased Brain Activity During Self-Referential Processing

Group differences in neural areas recruited during the presentation of emotional self-referential stimuli were found. Many more areas, including the bilateral inferior frontal gyrus and left middle temporal gyrus, were recruited during positive word processing (positive>neutral) than during negative self-referential word processing in social anxiety disorder. An increasing number of studies have proposed the fear of positive evaluation in social anxiety in addition to the fear of negative evaluation [10,40]. The increased inferior frontal gyrus and middle temporal gyrus activation reported herein further support previous findings that both regions are responsible for the down-regulation of socially driven emotions [41]. In particular, the left inferior frontal gyrus is well-known to be related to high selection demand among competing alternatives [42] as well as to self-referential processing [43]. This may indicate the possibility that the processing of positive self-referential information is a demanding task for socially anxious individuals.

Additionally, as hypothesized, negative self-referential processing (negative>neutral) was associated with stronger activation of the left insula in social anxiety disorder. This hyperactivation seems to reflect the aversive response or hypervigilance to negative self-referential stimuli [44-46].

### VR Therapy

#### Changes in Neural Responses to Positive Words

Another overarching aim of this fMRI study was to identify neural changes associated with self-referential processing as well as anxiety symptom reduction in social anxiety disorder following VR therapy designed to teach self-assertiveness. Because the control group did not participate in the therapy sessions, it cannot be decisively stated that the fMRI data obtained were the direct results of the VR therapy intervention. What is clear, however, is that there were significant changes in brain activity during positive self-referencing processing in the social anxiety disorder group who completed the VR sessions. That is, the participants showed not only significant reductions in social anxiety disorder symptoms but also neural changes in the right posterior cingulate cortex/precuneus, lingual gyrus, left inferior temporal gyrus, postcentral gyrus, and right precentral gyrus during positive self-referential processing after VR therapy.

We found increased activity in the posterior cingulate cortex/precuneus of individuals with social anxiety disorder. These cortical regions, along with the precentral gyrus, exhibit enhanced activation during the processing of positive self-referential stimuli in healthy volunteers [30]. The posterior cingulate cortex/precuneus also plays a crucial role in integrating autobiographical information regarding the self [47]. Interestingly, a recent finding from experimental research suggested that social anxiety relates to impaired memory for social scenarios that ended with positive outcomes [48]. Altogether, our results may reflect increased cognitive efforts to access autobiographical memory while processing positive self-referential information in social anxiety disorder. This result needs to be further studied using an autobiographical memory task and comparison with a control group.

After VR therapy, the activation of the lingual gyrus and inferior temporal gyrus during positive self-referential processing also increased. According to previous studies [49,50] using healthy populations, stronger activations of the lingual gyrus, inferior temporal gyrus, and posterior cingulate cortex are closely related to self-referential processing. Intriguingly, the greater the change in lingual gyrus activation while processing positive self-referential information, the lower the tendency to ruminate on negative life events and the lower the social anxiety levels following the VR intervention session. Additionally, the inferior temporal gyrus has been reported to play an important role, along with the posterior cingulate cortex, in judging whether a series of stimuli is self-related [51]. Therefore, increased BOLD signals in these cortical regions may reflect the facilitative effect of VR therapy on the process by which the individuals with social anxiety disorder accept the positive-valence words as being relevant to them.

The precentral and postcentral gyri also showed increased activation during positive self-referential processing in individuals with social anxiety disorder upon completing the VR sessions. A similar brain activation pattern was observed in individuals with anxiety disorders who used cognitive reappraisal [52]. We conjecture that activation in the postcentral gyrus represent the therapeutic effects of the social anxiety–focused VR program. Further research is needed to investigate whether the therapeutic effect of our VR program could be extended to individuals with other clinical conditions that exhibit reduced postcentral gyrus activity, such as those with a generalized anxiety disorder [53] or a history of childhood abuse [54].

#### Changes in Neural Responses to Negative Words

Participants with social anxiety disorder exhibited greater activation in cortical and subcortical regions, which are known to be involved in somatosensory integration, during the processing of negative self-referential stimuli after VR therapy than at baseline. Similar findings were reported from an fMRI study [19,55] showing the neural mechanisms of the cognitive reappraisal of negative self-beliefs in individuals diagnosed with social anxiety disorder; in particular, the participants with social anxiety disorder demonstrated greater cognitive and somatosensory brain responses while reappraising negative self-beliefs. The most recent study using healthy adults [56] showed that cognitive bias modification for interpretation resulted in significantly greater activations of the somatomotor and somatosensory areas and occipital lobe. Our results may suggest that the VR-based therapy facilitated the perceptual or sensory-motor processing of negative self-referential stimuli in individuals with social anxiety disorder who had been willing to avoid negative feedback cues. Further research using a control group is needed to explore whether such an enhancement of brain activation can be linked to an approach-oriented strategy with negative-valence stimuli in social anxiety disorder.

### Limitations

Several limitations should be noted. First, the case-control study using sham was not properly performed. Therefore, there is a limitation in interpretation to determine the effect of VR therapy through this study. Second, the lack of a direct comparison with conventional face-to-face therapy should be considered when interpreting these findings. A randomized controlled trial with a larger sample size is necessary to confirm the benefits of this VR therapy intervention on self-referential processing in social anxiety disorder. Finally, it should be noted that some our showed patterns similar to those of previous studies of depression [57]. In future research, a transdiagnostic approach would be a reasonable way to explore a variety of diagnostic criteria for other conditions.

### Conclusions

The body of literature supporting the effect of VR-based therapy for individuals with social anxiety disorder has been growing. Our study showed inefficient neural activation in a wide range of cortical regions, suggesting an increased predisposition for excessive cognitive processing in response to self-referential stimuli in social anxiety disorder. Following successful treatment with a VR intervention, symptoms in individuals with social anxiety disorder were reduced, which was demonstrated to be related to brain activation changes. Enhanced activations were also exhibited across brain regions that engage in self-image construction, autobiographical memory processing, and sensory information integration in healthy adults [30,47,56], indicating that VR may modulate the neural mechanisms responsible for self-reference. To our knowledge, this is the first neuroimaging study to specify the changes in the psychophysiological responses to self-referential information in social anxiety disorder in response to VR therapy. We believe that our findings may contribute to a better understanding of the therapeutic effects of VR-based interventions, which could be included in the routine treatment of social anxiety disorder.

## Abbreviations

ANOVA: analysis of variance
BOLD: blood oxygen level dependent
fMRI: functional magnetic resonance imaging
MANOVA: multivariate analysis of variance
MRI: magnetic resonance imaging
PERS: Post-Event Rumination Scale
PRRT: Personal Relevance Rating Task
SPS: Social Phobia Scale
VR: virtual reality
